# Multi-Omics Integrative Bioinformatics Analyses Reveal Long Non-coding RNA Modulates Genomic Integrity via Competing Endogenous RNA Mechanism and Serves as Novel Biomarkers for Overall Survival in Lung Adenocarcinoma

**DOI:** 10.3389/fcell.2021.691540

**Published:** 2021-07-22

**Authors:** Zhonglin Wang, Ziyuan Ren, Rui Li, Junpeng Ge, Guoming Zhang, Yaodong Xin, Yiqing Qu

**Affiliations:** ^1^Department of Pulmonary and Critical Care Medicine, Qilu Hospital, Cheeloo College of Medicine, Shandong University, Jinan, China; ^2^School of Physical Science, University of California, Irvine, Irvine, CA, United States; ^3^Department of Biology Engineering, Shandong Jianzhu University, Jinan, China; ^4^School of Statistics and Management, Shanghai University of Finance and Economics, Shanghai, China

**Keywords:** genome instability, lung adenocarcinoma, lncRNA, bioinformatics, ceRNA, prognosis

## Abstract

Long non-coding RNA (lncRNA) plays a crucial role in modulating genome instability, immune characteristics, and cancer progression, within which genome instability was identified as a critical regulator in tumorigenesis and tumor progression. However, the existing accounts fail to detail the regulatory role of genome instability in lung adenocarcinoma (LUAD). We explored the clinical value of genome instability-related lncRNA in LUAD with multi-omics bioinformatics analysis. We extracted the key genome instability-related and LUAD-related gene modules using weighted gene co-expression network analysis (WGCNA) and established a competing endogenous RNA (ceRNA) network using four lncRNAs (*LINC01224*, *LINC00346*, *TRPM2-AS*, and *CASC9*) and seven target mRNAs (*CCNF*, *PKMYT1*, *GCH1*, *TK1*, *PSAT1*, *ADAM33*, and *DDX11*). We found that *LINC01224* is primarily located in the cytoplasm and that *LINC00346* and *TRPM2-AS* are primarily located in the nucleus (*CASC9* unknown). We found that all 11 genes were positively related to tumor mutational burden and involve drug resistance, cancer stemness, and tumor microenvironment infiltration. Additionally, an eight-lncRNA genome instability-related lncRNA signature was established and validated, predicting the overall survival and immunotherapy outcomes in LUAD. To conclude, we discovered that sponging microRNA, genome instability-related lncRNA functions as ceRNA, modulating genomic integrity. This research provides clinical references for LUAD immunotherapy and prognosis and interprets a potential genome instability-related ceRNA regulatory network in which *LINC01224-miR-485-5p*/*miR-29c-3p-CCNF*-*RRM2* and *LINC01224-miR485-5p-PKMYT1*-*CDK1* axes were the most promising pathways. However, the potential mechanisms underlying our findings still need biological validation through *in vitro* and *in vivo* experiments.

## Introduction

At present, the incidence and mortality of lung cancer rank first among malignant tumors worldwide. Non-small-cell lung cancer (NSCLC) is the most common pathological type of lung cancer, accounting for 85% of all tumors, among which lung adenocarcinoma (LUAD) is the primary subtype, accounting for up to 40% (Zhong et al., 2019; Li et al., 2020; Niu et al., 2020). Unlike lung squamous cell carcinoma (LUSC), women and non-smokers comprise most LUAD patients (Pros et al., 2020). Besides, blood metastasis can occur in the early stage of LUAD without clinical symptoms, leading to a poor prognosis (Wang et al., 2019). Even though traditional therapeutic strategies, including surgery, chemoradiotherapy, and targeted molecular therapy, have rapidly developed in recent years, LUAD patients’ overall survival (OS) does not improve considerably (Lee et al., 2019). Current clinical practice has demonstrated the prominent effect of immunotherapy, especially after the discovery of immune checkpoint, primarily including programmed cell death 1 (*PD-1*), programmed cell death-ligand 1 (*PD-L1*), and cytotoxic T lymphocyte antigen 4 (*CTLA-4*). A recent prospective randomized clinical trial, KEYNOTE-042, demonstrated that pembrolizumab achieved a significantly longer OS in advanced LUAD patients even with low PD-L1 expression (Mok et al., 2019). Nevertheless, immune checkpoint inhibitor (ICI) expense significantly burdens patients and government health insurance (Carbone et al., 2017; Niu et al., 2020), and the median objective response rate is only 48.5% in PD-L1-overexpressing (≥50%) NSCLC (Frost et al., 2021). In addition to PD-L1, microsatellite instability (MSI) has been proved to be a novel ICI biomarker for colorectal cancer (Overman et al., 2017), but due to its rarity in LUAD, MSI cannot become promising ICI biomarkers for LUAD (Takamochi et al., 2017). Therefore, there is still an urgent need to explore more effective immunotherapy biomarkers.

It has previously been observed that tumor mutational burden (TMB) is closely related to an increased tumor immunotherapy response rate in LUAD and other cancer types (Rizvi et al., 2018; Liu et al., 2019; Yu et al., 2019). Genome instability, demonstrated as the endogenous source of mutation and tumor heterogeneity, modulates the alterations of epigenomic features (Tubbs and Nussenzweig, 2017; Peyraud and Italiano, 2020). Recent evidence suggests that long non-coding RNA (lncRNA) plays a critical role in regulating genome instability (Lee et al., 2016; Ventura, 2016; Hu et al., 2018; Munschauer et al., 2018). *NORAD* is the first discovered lncRNA, which assembles a critical topoisomerase complex maintaining genomic integrity. Hu et al. (2018) found that lncRNA *GUARDIN* functions as a scaffold RNA to sustain breast cancer 1 (BRCA1) stability. Moreover, *GUARDIN* could maintain chromosome end-to-end fusion through the *GUARDIN*-*miR-23*/*TRF2* pathway. However, the clinical value and potential regulatory mechanism of genome instability-related lncRNA in LUAD remain elusive.

Here, we performed an integrative multi-omics analysis to explore the mechanisms and clinical value of genome instability-related lncRNA (GlncR) in LUAD. We adopted a weighted gene co-expression network analysis (WGCNA) to extract the key genome instability-related and LUAD-related modules and then screened four lncRNAs (*LINC01224*, *LINC00346*, *TRPM2-AS*, and *CASC9*) that were most related to genome instability. We established a competing endogenous RNA (ceRNA) network and used subcellular localization analysis to investigate the potential regulatory role of the four lncRNAs. We applied TMB, MSI, drug sensitivity, immune analyses on the four lncRNAs and seven target messenger RNAs (mRNAs) (*CCNF*, *PKMYT1*, *GCH1*, *TK1*, *PSAT1*, *ADAM33*, and *DDX11*). To elucidate the clinical value of the GlncRs, we constructed an eight-lncRNA prognostic signature predicting the OS. Surprisingly, our signature could predict the response rate to PD-1/PD-L1 inhibitors, and several existing biomarkers (TMB, *PD-L1* expression, *POLE* mutation rate, and CD8+ cell infiltration) proved this point. To conclude, we established a novel genome instability-related ceRNA network and proposed an eight-lncRNA gene signature predicting OS and immunotherapy outcomes in LUAD.

## Materials and Methods

### Data Collection

The graphical presentation and online resources of our study design are shown in the graphical abstract. Clinical features, transcriptome profiling (gene expression quantification (RNA-seq, which was preprocessed by fragments per kilobase of an exon model per million mapped fragments (FPKM)) and microRNA (miRNA) expression quantification (miRNA-seq)), and simple nucleotide variation (SNV, Masked Somatic Mutation detected by VarScan 2) from the LUAD project of The Cancer Genome Atlas (TCGA) database were download through the Genomic Data Commons Data Portal website (https://portal.gdc.cancer.gov/, 2020.12.25). RNA-seq included 535 tumor samples and 59 paired normal samples. Corresponding clinical features included 500 patients (13 tumor samples were excluded due to lack of OS, and 22 tumor samples were duplicate samples (the average expression data of duplicate samples from one patient were utilized)). We used the “limma” package to normalize the transcriptome profiling (log2(FPKM + 1)). To identify the lncRNA and mRNA from total RNA-seq, the genome annotation file Genome Reference Consortium Human Build 38 patch release 13 (GRCh38.p13, downloaded from the National Center for Biotechnology Information)^1^ was used to reannotate the RNA-seq. We randomly divided the 500 samples into a training cohort (250 samples) and a validation cohort (250 samples) to build and validate the lncRNA prognostic signature. A chi-square test was utilized to detect the selection bias. The miRNA-seq was normalized by the “edgR” package, including 521 tumor samples and 46 paired normal samples. Extraction of the mutation status of each patient from SNV data was fulfilled by Perl language. Each patient’s TMB/MSI status was retrieved from the open-access bioinformatics website cBioPortal (the MSIsensor score was used, https://www.cbioportal.org/, 2020.12.26) (Cerami et al., 2012). Since all the data are open access, no ethics approval was acquired. The policies and publication guidelines of the TCGA database were strictly followed.

### WGCNA

We performed WGCNA by R package “WGCNA” to identify the gene co-expression modules that were most relevant to LUAD development and genome instability. The total RNA-seq, including 535 tumor samples and 59 normal samples, was included in the WGCNA of LUAD development. We first used R package “limma” to extract differentially expressed genes (DEGs) between the tumor sample and normal sample (adjusted *P*-value (adj.*P*) < 0.05; | log2 fold change (FC)| > 0.5) for the co-expression module construction. Six was set as the soft power using the pickSoftThreshold function. As for the WGCNA of LUAD genome instability, we first sorted all patients according to somatic mutation counts (SMC) from largest to smallest. The first quarter of patients was classified into the genome-unstable group (GU group). The last quarter of patients was classified into the genome-stable group (GS group). Only the RNA-seq of the GS and GU groups was included in the WGCNA of LUAD genome instability. Then, we extracted the DEGs between the GS and GU groups (adj.*P* < 0.05; | log2 FC| > 0.5) for the co-expression module construction. Five was set as soft power. The detailed steps of the WGCNA technology followed the “WGCNA” package instruction. The code we used could be acquired by contacting the corresponding author.

### Identification of Genome Instability-Related lncRNA (GlncRs), mRNA (GmRs), and miRNA (GmiRs)

We used R package “limma” to extract DEGs between the GU and GS groups. Differentially expressed lncRNAs were named GlncRs (adj.*P* < 0.05; | log2 FC| > 1), mRNAs were named GmRs (adj.*P* < 0.05; | log2 FC| > 1), and miRNAs were named GmiRs (adj.*P* < 0.05; | log2 FC| > 0.5).

### ceRNA Network Construction

The overlapping lncRNAs in the top three LUAD development-related modules, genome instability-related module, and GlncRs were included in the ceRNA network construction. We searched an online ceRNA interaction network predictive database, ENCORI (The Encyclopedia of RNA Interactomes, ceRNA interaction network, http://starbase.sysu.edu.cn/ceRNA.php?source=lncRNA, 2021.1.12), for each included lncRNA to explore the possible target mRNAs (Li et al., 2013). As described on the website, the presented ceRNA interactive network from thousands of interactions of miRNA–targets was supported by crosslinking immunoprecipitation (CLIP)-seq data. We then excluded the GlncR target mRNAs that are *neither* in the module most related to genome instability *nor* in the GmRs. Furthermore, only the lncRNA–mRNA pairs that are statistically significant (*P* < 0.05) in LUAD based on the ENCORI database (through a pan-cancer analysis of miRNA–targets and RBP (RNA-binding protein)-RNAs in 32 types of cancers) remained. Then, the predicted mediating miRNAs between the lncRNA–mRNA pairs were retrieved. The overlapping miRNAs in GmiRs and predicted miRNAs were included in the ceRNA networks. The graphical representation of the screening process is presented in Figures 2A–C. Then, to explore the potential function of the included lncRNAs, an online lncRNA subcellular localization database, lncATLAS,^2^ was utilized. Cytoscape, a software for processing complicated networks, was used to modify the ceRNA network (Shannon et al., 2003).

### Genome Instability, Immune, Drug Sensitivity, and Cancer Stemness Analyses

We performed a pan-cancer analysis to explore the potential biological function of the lncRNAs and their target mRNAs in the ceRNA network based on TCGA pan-cancer data downloaded from the Xena platform,^3^ including RNA-seq, clinical data, and cancer stemness scores based on mRNA expression (RNA stemness score, RNAss) and DNA methylation pattern (DNA stemness score, DNAss) (Malta et al., 2018). Thirty-three cancer types (ACC, BLCA, BRCA, CESC, CHOL, COAD, DLBC, ESCA, GBM, HNSC, KICH, KIRC, KIRP, LAML, LGG, LIHC, LUAD, LUSC, MESO, OV, PAAD, PCPG, PRAD, READ, SARC, SKCM, STAD, TGCT, THCA, THYM, UCEC, UCS, and UVM) were included. We calculated the correlation coefficient between each gene and the TMB/MSI status in 33 cancer types by Spearman correlation. We then calculated the correlation coefficient between every two genes by Spearman correlation to detect these genes’ interactions. Genome instability fosters tumor heterogeneity, contributing to tumor progression, drug resistance, tumor microenvironment (TME) alterations, and cancer stemness (Morel et al., 2017; Dagogo-Jack and Shaw, 2018; Sansregret et al., 2018). Therefore, the prognosis, drug sensitivity, TME infiltration, and cancer stemness analysis of these genes were conducted. GEPIA,^4^ an online website bioinformatics tool, was utilized to perform survival analysis based on overall analysis and disease-free survival (DFS). We utilized the National Cancer Institute (NCI)-60^5^ database to perform the drug sensitivity analysis of the lncRNAs and their target mRNAs. NCI-60 is an open-access database based on nine cancer types and 60 cancer cell lines, consisting of mRNA expression level and corresponding *z* scores of cell sensitivity data (GI50) after drug treatment. We calculated the Pearson correlation between each gene expression and the GI50 to explore the association between these genes and drug sensitivity. We selected 262 FDA-approved drugs or drugs that are currently in clinical trials in this drug sensitivity analysis (Zhang et al., 2020). TME infiltration analysis was performed by the Estimation of STromal and Immune cells in MAlignant Tumor tissues using Expression data (ESTIMATE) immune and stromal score downloaded from ESTIMATE^6^ (Yoshihara et al., 2013). Furthermore, to validate these genes’ immune function, the six immune subtypes obtained from TCGA pan-cancer data were used to test the association between each gene and immune infiltrate types by analysis of variance (Thorsson et al., 2018). The cancer stemness features obtained from TCGA pan-cancer data were used to test the association between each gene and stem-cell-like features of tumor cells by Spearman correlation test. To further explore these genes’ potential mechanisms in modulating genomic integrity and TME, we detected the correlation between each gene and four MMR genes (*MLH1*, *MSH2*, *MSH6*, and *PMS2*), six immune-checkpoint-related genes (*PD-L1* (*CD274*), *PDCD1*, *PDCD1LG2*, *CTLA4*, *CD80*, and *CD86*), and two previously discovered genome instability regulatory lncRNAs (*NORAD* (Munschauer et al., 2018) and *LNCTAM34A* (*GUARDIN*) (Hu et al., 2018)). The normality test of the indexes (TMB/MSI score, drug sensitivity index, ESTIMATE score, and RNAss/DNAss) was performed by the Kolmogorov–Smirnov test (Supplementary Table 1). The drug sensitivity indexes were normally distributed. Therefore, the method of Pearson correlation between gene expression and drug sensitivity was reasonable.

### Construction and Validation of the Genome Instability-Related lncRNA Prognostic Signature (GIRlncPS)

We used the uni- and multi-variate Cox regression to construct the GIRlncPS based on OS in the training cohort using GlncRs. The risk score was calculated as follows: risk score = sum(coefficient (multivariate Cox regression) × corresponding lncRNA expression). Hazard ratio (HR) was calculated as exp(coefficient). Dividing the training cohort based on the median risk score, we classified all 500 patients into high- and low-risk groups. Survival analysis by the Kaplan–Meier (KM) curve and the log-rank test was conducted to evaluate the prognostic value of the GIRlncPS. The receiver operating characteristic (ROC) curve and area under the curve (AUC) were utilized to evaluate the reliability of the GIRlncPS. The TMB status, MSI status, expression of four MMR genes (*MLH1*, *MSH6*, *PMS2*, and *MSH2*), two immune-checkpoint-related genes (*CD274* (*PD-L1*) and *CTLA4*), and the mutation rate of *POLE* (a novel discovered biomarker for ICI therapy outcomes (Wang F. et al., 2019)) of the two groups were compared by the Mann–Whitney *U* test. The R package “gsva” was utilized to perform the single-sample gene set enrichment analysis (ssGSEA) to compare the two groups’ immune cell infiltration and immune functions. Furthermore, we compared the mutation rate of *TP53* between the two groups in the TCGA cohort. *TP53* is one of the most mutated tumor suppressor genes acting as a genomic integrity guard. We classified the patients into four groups, *TP53* mutated/high-risk, *TP53* mutated/low-risk, *TP53* wild/high-risk, *TP53* wild/low-risk groups, to detect if the GIRlncPS has better predictive ability than *TP53*. Survival analysis by the KM curve between the four groups was performed. Then, we searched Google Scholar for previously published prognostic lncRNA signatures for LUAD. ROC curve and AUC were used to compare these signatures’ predictive ability with the GIRlncPS based on OS at 1, 2, and 3 years in the overlapping patients (500 patients). The included signatures were as follows: Li’s signature, 2020, and Sui’s signature, 2020. Independent analysis with clinical prognostic factors was performed by uni- and multi-variate Cox regression in the TCGA cohort. The included clinical features were as follows: age (>65 vs. ≤65), gender (male vs. female), stage (III/IV vs. I/II), T (III/IV vs. I/II), M (I vs. 0), N (I/II vs. 0), *EGFR* (mutation vs. wild), and *TP53* (mutation vs. wild). We plotted the nomogram in the TCGA cohort for clinical reference. The calibration curve was plotted to evaluate the reliability of the nomogram.

### Statistics

The descriptive analysis and normality test were conducted by IBM SPSS Statistics 26.0 (International Business Machines Corporation, Armonk, NY, United States). Other statistics were performed by R language (version 4.0.3) (R Core Team, 2013). The adj.*P* (*q*-value and false discovery rate (FDR)) was adjusted by Benjamini and Hochberg. Adj.*P* < 0.05 was considered statistically significant in DEG extraction, and *P*-value < 0.05 was significant in other conditions.

## Results

### WGCNA

**FIGURE 1:**
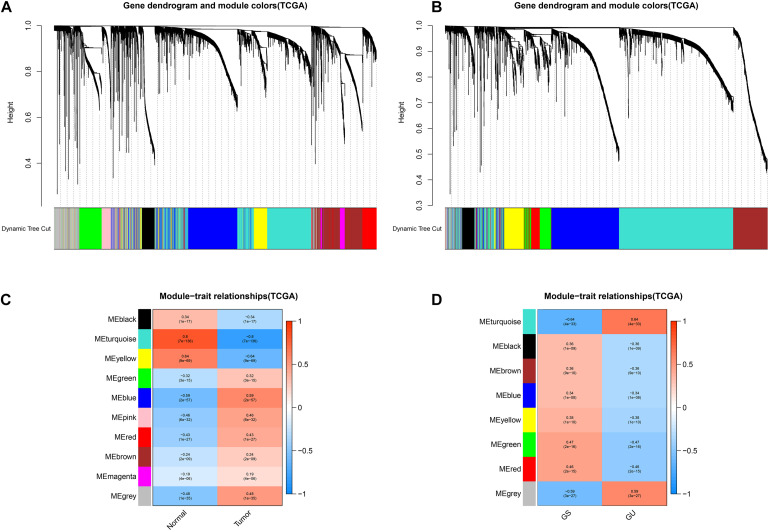
Identification of key tumor- and genome instability-related modules via the WGCNA. The cluster dendrogram of genes in the key tumor-related modules **(A)** and genome instability-related modules **(B)**. The module–trait relationship of key tumor-related modules **(C)** and genome instability-related modules **(D)**. The coefficient varies from –1 to 1 as color changes as blue–white–red. GS, genome stable; GU, genome unstable.

We utilized the “WGCNA” to explore the potential gene modules with the closest relation to LUAD development and genome instability. The results of the WGCNA are presented in Figure 1. As for the WGCNA of LUAD development, 5,326 genes were included in the co-expression module construction, and a total of 10 gene co-expression modules were acquired. The top three LUAD development-related modules were turquoise (*r* = 0.8, *P* = 7e−136), yellow (*r* = 0.64, *P* = 9e−69), and blue modules (*r* = 0.59, *P* = 2e−57), indicating that the genes cooperate to promote tumorigenesis in these three modules, respectively. Similarly, a total of 3,489 genes were included in the WGCNA of LUAD genome instability. As shown in Figures 1B,D, eight modules were acquired, and the turquoise module was most relevant to genome instability (*r* = 0.64, *P* = 4e−33). The detailed results, including the gene significance (GS) and module membership (MM) of each gene, are provided in Supplementary Tables 2 and 3.

**FIGURE 2:**
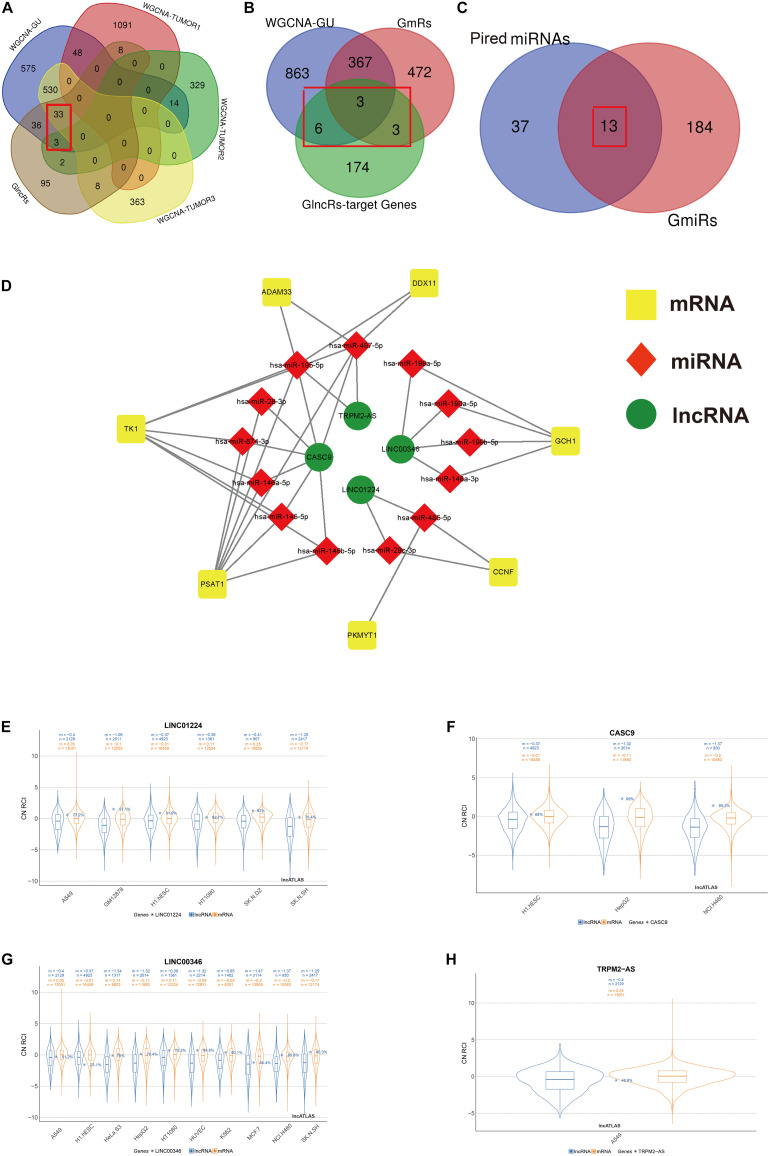
Screening process of the included lncRNA, mRNA, and miRNA and construction of genome instability-related ceRNA network. The Venn diagrams present the screening process of included lncRNA **(A)**, mRNA **(B)**, and miRNA **(C)** by WGCNA gene modules that are the most relevant to both genome instability and tumorigenesis and differential expression analysis between GS samples and GU samples. **(D)** The ceRNA network based on screened lncRNA, mRNA, and miRNA using an online database ENCORI. **(E–H)** The subcellular localization of *LINC01224*, *CASC9*, *LINC00346*, and *TRPM2-AS* based on the lncATLAS database. “n”, the number of total genes; “m”, the mean value of total gene expressions. Blue, mRNA. Orange, lncRNA. GU, genome unstable. GlncRs, genome instability-related lncRNAs; GmRs, genome instability-related mRNAs; GmiRs, genome instability-related miRNAs; RCI, relative concentration index; CN, cytoplasmic/nuclear.

### Identification of GlncRs, GmRs, and GmiRs and ceRNA Network Construction

We used R package “limma” to extract genome instability-related genes between the GU and GS groups. In total, 185 GlncRs, 845 GmRs, and 197 GmiRs were obtained. ceRNA is a common regulatory mechanism of lncRNA. Salmena et al. (2011) first proposed the ceRNA mechanism, describing that RNA transcripts containing numerous miRNA-binding sites could competitively sponge miRNA, altering the miRNA target genes’ function (Tay et al., 2014). Because we hope to extract the genome instability-related lncRNA–miRNA–mRNA pairs precisely, a rigorous screening process is presented in Figures 2A–C. Given that WGCNA was used to extract key gene modules related to genome instability and tumorigenesis, we first intersected the module that was most related to genome instability, the top three modules that were most related to tumorigenesis, and the GlncRs to screen the crucial lncRNAs (we found 36 lncRNAs) that are related to genome instability and LUAD tumorigenesis. Then, we utilized an online database (ENCORI) to explore the potential mRNAs (GlncR target mRNAs) of these 36 lncRNAs. We excluded the GlncR target mRNAs that are *neither* in the module most related to genome instability *nor* in the GmRs. Further, only the lncRNA–mRNA pairs that are statistically significant (*P* < 0.05) in LUAD based on the ENCORI database (through a pan-cancer analysis of miRNA–targets and RBP (RNA-binding protein)-RNAs in 32 types of cancers) remained. The remaining 12 mRNAs were considered the crucial mRNAs that were most related to genome instability. Last, we intersected the predicted miRNAs (based on ENCORI) and the GmiRs (we found 13 miRNAs). Meanwhile, some mRNAs in the 12 mRNAs were excluded because their paired miRNAs were not included in the 13 miRNAs. Finally, we only got four lncRNAs, seven mRNAs, and 13 miRNAs. The detailed retrieved information about the 36 lncRNAs from the ENCORI database and lncRNA–miRNA–mRNA pairs before being screened by crucial mRNA and miRNA is provided in Supplementary Tables 4 and 5. Table 1 displays the ceRNA network. The modified graphical ceRNA network is presented in Figure 2D.

**TABLE 1:** Genome instability-related ceRNA network.

lncRNA	miRNA	mRNA
TRPM2-AS	hsa-miR-195-5p	DDX11
TRPM2-AS	hsa-miR-497-5p	DDX11
TRPM2-AS	hsa-miR-195-5p	ADAM33
TRPM2-AS	hsa-miR-497-5p	ADAM33
LINC01224	hsa-miR-485-5p	PKMYT1
LINC01224	hsa-miR-485-5p	CCNF
LINC01224	hsa-miR-29c-3p	CCNF
LINC00346	hsa-miR-148a-3p	GCH1
LINC00346	hsa-miR-190a-5p	GCH1
LINC00346	hsa-miR-199a-5p	GCH1
LINC00346	hsa-miR-199b-5p	GCH1
CASC9	hsa-miR-145-5p	TK1
CASC9	hsa-miR-146a-5p	TK1
CASC9	hsa-miR-874-3p	TK1
CASC9	hsa-miR-146b-5p	TK1
CASC9	hsa-miR-195-5p	TK1
CASC9	hsa-miR-497-5p	TK1
CASC9	hsa-miR-145-5p	PSAT1
CASC9	hsa-miR-146a-5p	PSAT1
CASC9	hsa-miR-28-3p	PSAT1
CASC9	hsa-miR-874-3p	PSAT1
CASC9	hsa-miR-146b-5p	PSAT1
CASC9	hsa-miR-195-5p	PSAT1
CASC9	hsa-miR-497-5p	PSAT1

It is reported that lncRNAs’ unique subcellular localization was closely associated with their functions and that cytoplasmic lncRNAs could serve as ceRNA (Chen, 2016). We utilized the lncATLAS database to investigate the subcellular localization of the four lncRNAs. A549 is the specific cell line for NSCLC. Among the four included lncRNA, *LINC01224* was mainly located in the cytoplasm in A549, and *LINC00346* and *TRPM2-AS* were mainly located in the nucleus in A549. The subcellular localization of *CASC9* in A549 was unclear, but mainly in the cytoplasm in other cell lines (Figures 2E–H). These results indicated that *LINC01224* and *CASC9* might primarily modulate genomic integrity via the ceRNA mechanism and that *LINC00346* and *TRPM2-AS* might primarily modulate genomic integrity via direct regulation.

### Genome Instability, Immune, Cancer Stemness, and Drug Sensitivity Analyses

To comprehensively understand these four lncRNAs (*LINC01224*, *CASC9*, *LINC00346*, and *TRPM2-AS*) and seven target mRNAs (*CCNF*, *PKMYT1*, *GCH1*, *TK1*, *PSAT1*, *ADAM33*, and *DDX11*), we performed genome instability, immune, cancer stemness, and drug sensitivity analysis.

**FIGURE 3:**
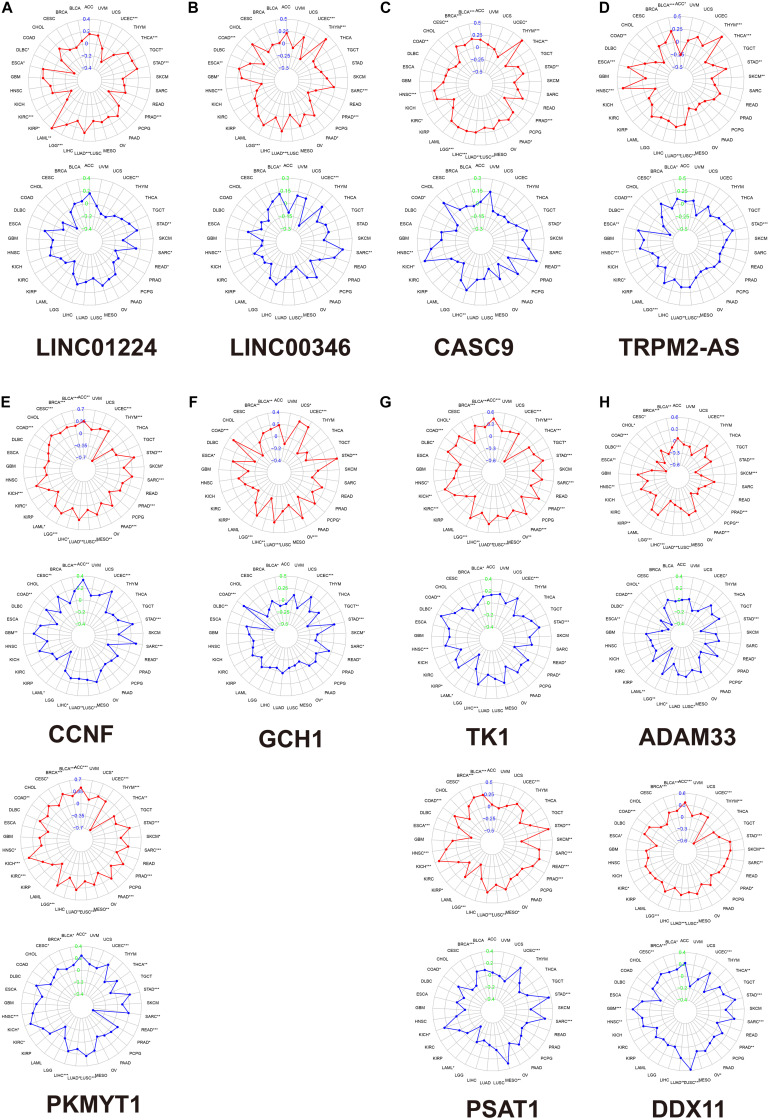
The association between each gene expression and TMB/MSI score based on the TCGA database by Spearman correlation. The red plot indicates TMB. The blue plot indicates MSI. **(A)** *LINC01224*. **(B)** *LINC00346*. **(C)** *CASC9*. **(D)** *TRPM2-AS*. **(E)** *LINC01224* target mRNAs (*CCNF* and *PKMYT1*). **(F)** *LINC00346* target mRNA (*GCH1*). **(G)** *CASC9* target mRNAs (*TK1* and *PSAT1*). **(H)** *TRPM2-AS* target mRNAs (*ADAM33* and *DDX11*). *, *P* < 0.05; **, *P* < 0.01; ***, *P* < 0.001. TMB, tumor mutation burden; MSI, microsatellite instability.

**FIGURE 4:**
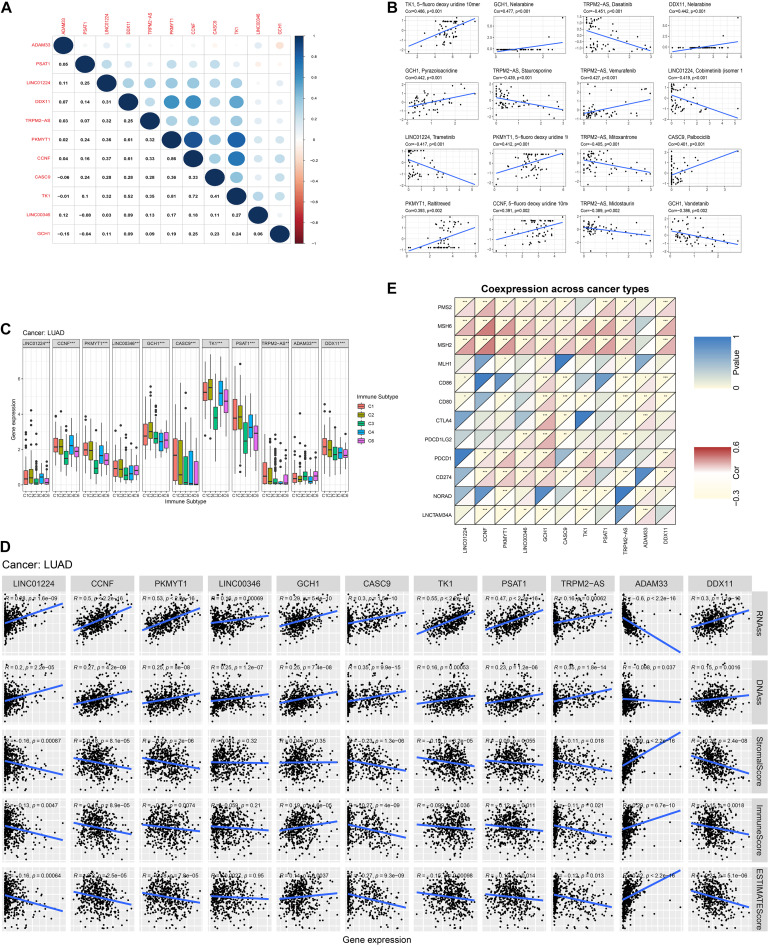
Pan-cancer analysis of the four lncRNAs and their seven target mRNAs. **(A)** The co-expression analysis between every two genes by Spearman correlation based on pan-cancer data. **(B)** The drug sensitivity analysis based on the NCI-60 database by Spearman correlation. **(C)** The relationship between each gene and immune subtype by the Kruskal–Wallis test in LUAD. **(D)** The co-expression analysis between each gene and four MMR genes (*MLH1*, *MSH2*, *MSH6*, and *PMS2*), six immune-checkpoint-related genes (*PD-L1* (*CD274*), *PDCD1*, *PDCD1LG2*, *CTLA4*, *CD80*, and *CD86*), and two previously discovered genome instability regulatory lncRNAs (*NORAD* and *LNCTAM34A* (*GUARDIN*)) by Spearman analysis in LUAD. **(E)** The relationship between each gene and tumor stemness indexes (RNA stemness score, RNAss; DNA stemness score, DNAss) and immune cell, stromal cell, and tumor purity (ESTIMATE score) in LUAD.

We calculated the Spearman correlation between each of the 11 gene expressions and the TMB and MSI score in pan-cancer. Figure 3 presents the results of genome-instability analysis of included lncRNAs and target mRNAs. Surprisingly, we found that all the 11 genes’ expression was significantly associated with TMB in LUAD (*P* < 0.001). Most (10/11, 90.9%) genes were positively related to the TMB, and only *ADAM33* was negatively related to the TMB (*r* = −0.18), indicating that *ADAM33* plays a crucial role in maintaining genomic integrity and that other genes contribute to genome instability in LUAD. Notably, among the four lncRNAs, *LINC00346* had the closest relation to TMB with a Spearman correlation exceeding 0.25. Among the seven target genes, the Spearman correlation between *CCNF*, *PKMYT1*, and *TK1* reached 0.35. Besides, we found that *TRPM2-AS*, *CCNF*, *PKMYT1*, and *DDX11* (4/11, 36.4%) were positively related to MSI score, indicating that these four genes function as key regulators in MMR mechanisms. We then calculated the correlation coefficient between every two genes by Spearman correlation to detect these genes’ interactions. The results were presented in Figure 4A. The correlation between *PKMYT1* and *DDX11* reached 0.61, that between *PKMYT1* and *CCNF* reached 0.86, that between *PKMYT1* and *TK1* reached 0.81, and that between *CCNF* and *TK1* reached 0.72, indicating these genes have a synergistic effect. Extensive research has shown that genome instability is one of the major causes of intratumoral heterogeneity, leading to drug resistance. The top 16 correlations between genes and drug sensitivity are shown in Figure 4B. It is shown that sensitivity of drugs that affect DNA replication and synthesis, such as 5-fluorodeoxyuridine 10mer, pyrazoloacridine, and palbociclib, was positively related to GlncRs and paired mRNA expression. These results further indicate the clinical usage of GlncRs. Furthermore, as for some protein kinase-targeted drugs, such as dasatinib and cobimetinib, genome instability-related genes were negatively related to the sensitivity. We then performed the immune analysis of these 11 genes. The results of immune subtype analysis in LUAD are shown in Figure 4C, which evaluates various immune functions of each gene related to lung cancer: wound healing (C1), IFN-γ dominant (C2), inflammatory (C3), lymphocyte depleted (C4), immunologically quiet (C5), and TGF-β dominant (C6) (Thorsson et al., 2018). *PKMYT1* in C1 had the highest expression among the five subtypes, corresponding to wound healing, and this may account for the increase of expression of angiogenic genes and the high proliferation rate. All genes were significantly related to immune subtypes, indicating that genome instability-related genes play a crucial role in tumor immunity. We performed survival analysis of the 11 genes based on OS and DFS using an online bioinformatics website tool GEPIA. The results were displayed in Supplementary Figure 1. The high expression of *PKMYT1*, *TK1*, and *LINC00346* was related to lower OS and DFS, indicating that these three genes could promote LUAD progression and serve as novel prognostic biomarkers. Besides, the high expression of *ADAM33* was related to lower OS. To explore potential mechanisms of genome instability-related genes in modulating genome integrity and tumor immune features, we calculated the Spearman correlation between the expression of the 11 genes and genome instability and immune-related genes (*PDCD1*, *CD274*, *PDCD1LG2*, *CTLA4*, *CD80*, *CD86*, *MSH2*, *MLH1*, *PMS2*, *MSH6*, *NORAD*, and *GUARDIN*) (Figure 4E). We found that most genes (10/11, 90.9%) were positively related to *MSH2* and *MSH6* (*P* < 0.01), indicating that high expression of MMR proteins was not consistent with high genomic integrity in LUAD. Most genes were positively related to *PD-L1* (*CD274*) (6/11, 54.5%) and *PD-1* (*PDCD1*) (7/11, 63.6%) (*P* < 0.01). Furthermore, five genes (*CCNF*, *PKMYT1*, *LINC00346*, *GCH1*, and *TK1*) were negatively related to *GUARDIN* (*LNCTAM334A*). These findings suggested that these 11 genes modulate the *GUARDIN* pathway and contribute to a predicted good ICI outcome. Furthermore, we utilized the ESTIMATE and CIBERSORT databases to explore LUAD TME infiltration. As shown in Figure 4D, most genes were negatively related to stromal cell infiltration (8/11, 72.7%) and immune cell infiltration (9/11, 81.8%) and positively related to tumor purity (9/11, 81.8%). Besides, as shown in Supplementary Figure 2, some critical immune cell infiltrations that affect immunotherapy, such as CD8+ T cells and active NK cells, were positively related to these genes’ expression (10/11, 90.9% for CD8+ cells; 9/11, 81.8% for active NK cells). Moreover, M2-type macrophage infiltration, which mainly exerts protumor effects, was negatively related to most genes’ expression (10/11, 90.9%), while M1-type macrophage infiltration, which mainly exerts antitumor and proinflammatory effects, was positively related to most genes’ expression (10/11, 90.9%). These findings indicated that the 11 genes we proposed contribute to an antitumor TME infiltration and are beneficial for immunotherapy. Given that genome instability is a crucial regulator for cancer stemness, we explored the association between gene expression and cancer stemness features (including RNAss and DNAss). The results showed that most genes were positively related to cancer stemness (10/11, 90.9%) (Figure 4D), among which the correlation between *CCNF*, *PKMYT1*, *TK1*, and RNAss reached 0.5 (*P* < 0.001). Noticeably, one mRNA, *ADAM33*, was different from the other 10 genes in most conditions, indicating that *ADAM33* involves other complex biological processes.

### Construction and Validation of the GIRlncPS

**FIGURE 5:**
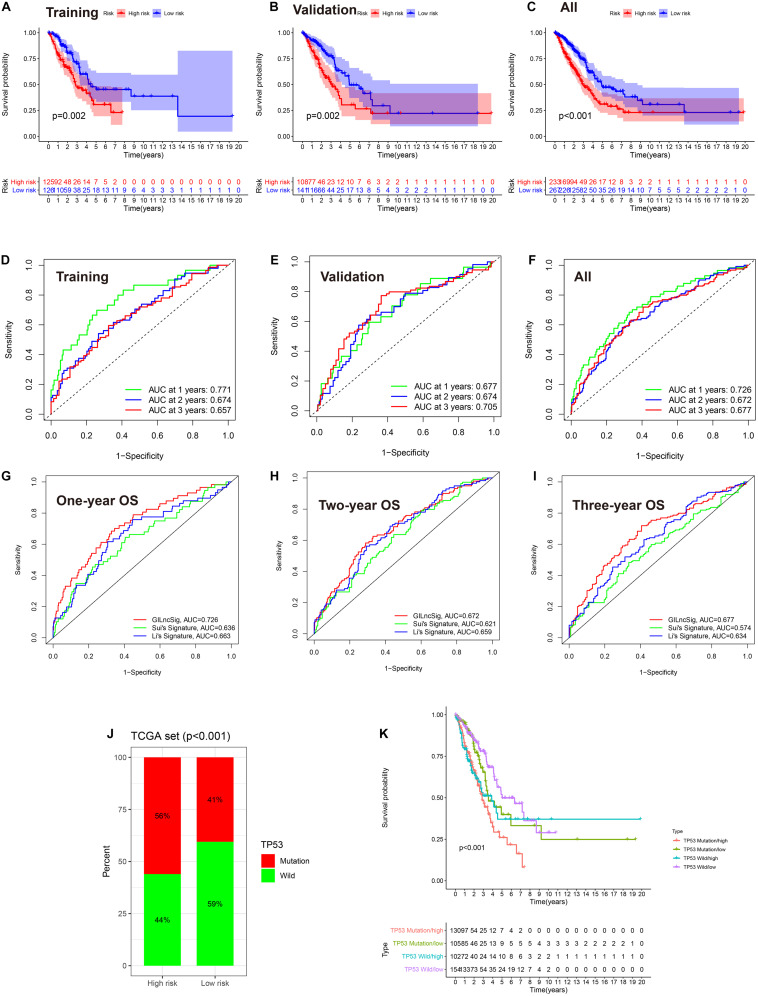
The eight-lncRNA prognostic signature based on the TCGA-LUAD cohort. **(A–C)** Survival analysis between the high- and low-risk groups based on OS. **(D–F)** ROC curve of the signature model based on OS at 1, 2, and 3 years. AUC, area under the curve. **(G–I)** Model comparison with two previously published lncRNA prognostic signatures by the ROC curve based on OS in the TCGA cohort at 1, 2, and 3 years. **(J)** The comparison of the mutation status of *TP53* between the high- and low-risk groups. **(K)** Survival analysis between *TP53* mutated/high-risk, *TP53* mutated/low-risk, *TP53* wild/high-risk, and *TP53* wild/low-risk groups by the Kaplan–Meier curve. **(A,D)** training cohort; **(B,E)** validation cohort; **(C,F)**, the entire TCGA cohort.

To explore the prognostic value of GlncRs in LUAD, we established a prognostic signature based on the 185 GlncRs. We randomly divided the 500 samples into a training cohort (250 patients) and a validation cohort (250 patients). The basic characteristics of the TCGA-LUAD cohort are presented in Supplementary Table 6. According to the chi-square test, there was no selection bias between the training and validation cohorts. We then performed uni- and multi-variate Cox regression in the training cohort, and the results are displayed in Table 2. The risk score was calculated as follows: risk score = sum(coefficient × corresponding lncRNA expression (log2(normalized gene expression + 1))). Hazard ratio (HR) was calculated as exp(coefficient). An eight-lncRNA GIRlncPS was acquired: 0.03 × FAM83A-AS1 + 0.05 × LINC02587 − 0.69 × MIR99AHG − 0.154 × AL07 8645.1 + 0.05 × LINC01671 + 0.03 × PLAC4 + 0.06 × LINC0 1511 + 0.09 × LINC01116. The predictive ability of our GIRlncPS in the training, validation, and whole TCGA cohorts was acceptable for lncRNA prognostic signature. Survival analysis indicated the OS was significantly longer in the low-risk group in the three cohorts (Figures 5A–C), and all the AUC reached 0.65 (Figures 5D–F). Compared with two previously published lncRNA prognostic signatures based on OS at 1, 2, and 3 years in LUAD, the GIRlncPS had the best prognostic ability (AUC = 0.726, 0.672, and 0.677, respectively) (Figures 5G–I). To explore whether the GIRlncPS had better prognostic ability than he *TP53* mutation, we analyzed the *TP53* mutation rate between the two groups. The *TP53* mutation rate in the high-risk group was 58%, significantly higher than that in the low-risk group (41%). We classified the patients into four groups: *TP53* mutated/high-risk, *TP53* mutated/low-risk, *TP53* wild/high-risk, *TP53* wild/low-risk groups. According to the survival analysis between the four groups, GIRlncPS had better prognostic ability than *TP53* (Figure 5K). Regardless of whether the *TP53* was mutated, the high-risk groups had a significantly lower OS. Independent analysis with clinical features by uni- and multi-variate Cox regression indicated older age, higher stage, T, and N, which were significant risk factors based on OS in the TCGA cohort, and the low-risk group was a significant protective factor (Supplementary Figures 3A,B). To provide a clinical reference for clinicians, a nomogram based on OS in the TCGA cohort was presented in Supplementary Figure 3C. The calibration curve indicated that the nomogram could perform well in predicting LUAD patients’ OS, especially at 2 and 3 years (Supplementary Figure 3D).

**TABLE 2:** The results of uni- and multi-variate Cox regression.

id	Univariate Cox regression				Multivariate Cox regression			
		
	HR	HR.95L	HR.95H	*P*-value	HR	HR.95L	HR.95H	*P*-value
AC012085.2	1.14	1.04	1.26	0.01				
FAM83A-AS1	1.04	1.01	1.06	0.00	1.03	1.01	1.06	0.02
LINC02587	1.04	1.02	1.07	0.00	1.05	1.02	1.07	0.00
LINC02864	1.12	1.02	1.22	0.01				
AL138760.1	1.08	1.00	1.17	0.04				
AC110619.1	1.20	1.01	1.42	0.04				
MIR99AHG	0.29	0.11	0.80	0.02	0.50	0.19	1.36	0.18
SCAT1	1.19	1.04	1.36	0.01				
AC003092.1	1.02	1.00	1.03	0.04				
AL078645.1	0.11	0.01	0.94	0.04	0.21	0.03	1.74	0.15
LINC01671	1.04	1.01	1.07	0.00	1.05	1.02	1.08	0.00
PLAC4	1.03	1.01	1.05	0.01	1.03	1.01	1.05	0.01
LINC01511	1.06	1.03	1.08	0.00	1.06	1.03	1.09	0.00
AC131009.1	1.40	1.01	1.94	0.04				
LINC01116	1.12	1.03	1.21	0.01	1.09	1.01	1.19	0.03

*HR, hazard ratio, calculated by exp(coefficient), 95L/R, 95% confidence interval.*

**FIGURE 6:**
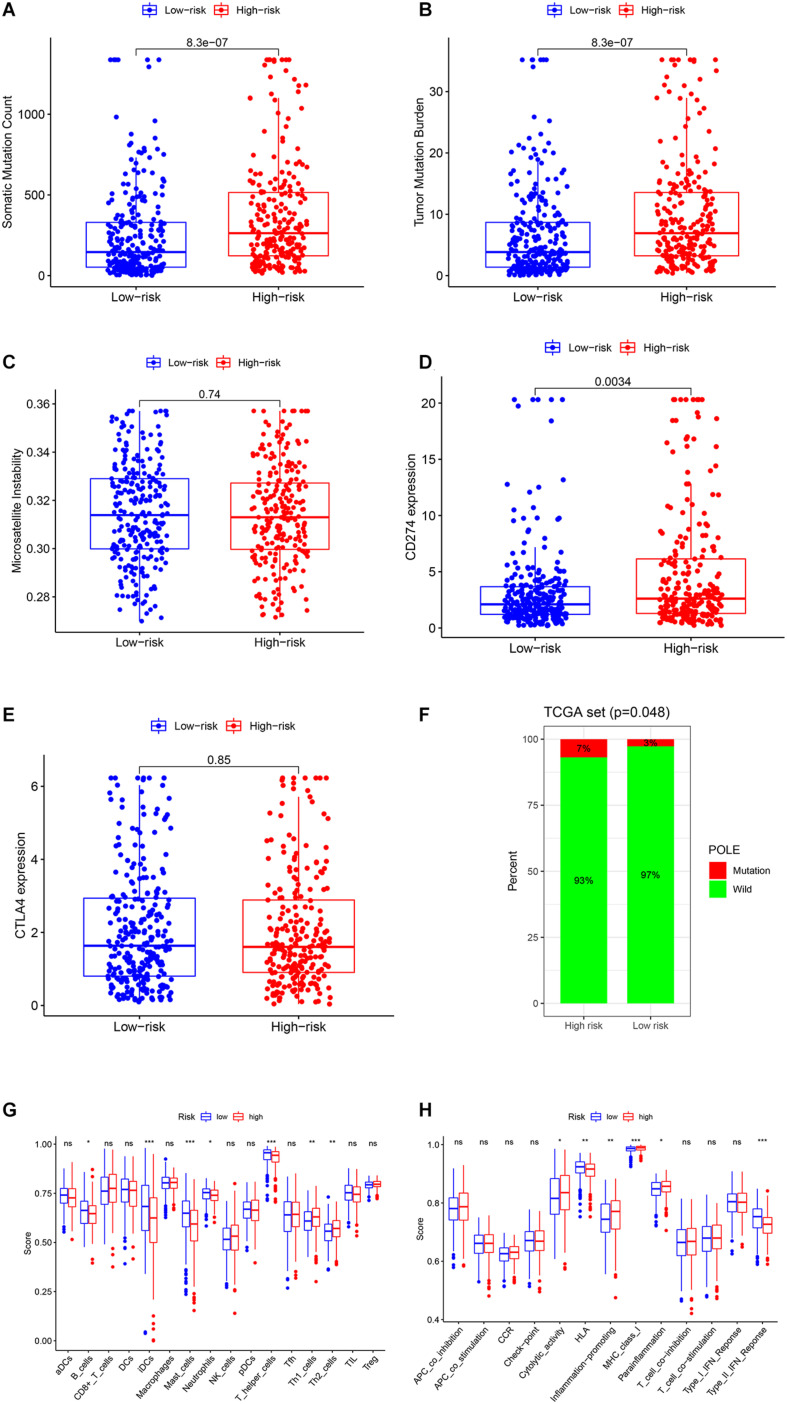
Genome instability and immune analysis of high- and low-risk groups. The comparison of the SMC **(A)**, TMB **(B)**, MSI **(C)**, the expression and mutation status of *CD274* (*PD-L1*) **(D)**, the expression of *CTLA4* **(E)**, and the mutation status of POLE **(F)** between the high- and low-risk groups. The comparison of the immune cell infiltration **(G)** and immune functions **(H)** of the two groups by ssGSEA. *, *P* < 0.05; **, *P* < 0.01; ***, *P* < 0.001.

We also performed the genome instability and immune analyses in the high- and low-risk groups. As shown in Figures 6A–C, both SMC and TMB were significantly higher in the high-risk group, while there was no difference between the MSI score of the two groups. The patients in the high-risk group had a higher *PD-L1* (*CD274*) expression and *POLE* mutation rate but not *CTLA4* expression (Figures 6D–F). Immune cell infiltration and immune function analysis indicated that most immune cell types, such as T helper cells, mast cells, and B cells, were significantly higher in the low-risk group. But some essential immune cells participating in immune checkpoint mechanisms, such as CD8+ cells and Th cells, were even higher in high-risk groups. Similarly, in immune function analysis, although some innate immune processes, such as type II IFN response, were lower in the high-risk group, cytolytic activity and antigen presentation process such as MHC class I were higher in the high-risk groups (Figures 6G,H). These results indicated that the high-risk group probably had a higher response rate to PD-1/PD-L1 inhibitors, and several existing biomarkers (TMB, *PD-L1* expression, *POLE* mutation rate, and CD8+ cell infiltration) proved this point.

## Discussion

Immunotherapy has achieved a gratifying breakthrough in lung cancer, especially NSCLC (Sun et al., 2020). Predominantly, ICIs aroused interest and led to a remarkable improvement of DFS and OS. Monoclonal antibodies targeting the CTLA-4 pathway (ipilimumab) (Rittmeyer et al., 2017), PD-1 (nivolumab and pembrolizumab) (Tanvetyanon et al., 2016), and PD-L1 (durvalumab, atezolizumab, and avelumab) (Zhou et al., 2016) have achieved promising improvements in second-line therapy for advanced lung cancers. ICIs enhance the intrinsic immune response against tumor antigens by eliminating the brake on T-cell activation by antigen-presenting cells. Despite substantive progress in lung carcinoma immunotherapy, the objective response rate in LUAD patients remains unsatisfying. The combination of standard immunotherapy and neoadjuvant therapy promoting immune system antitumor effects is an alluring strategy (Li F. et al., 2020). Tumor initiation and progression depend on the genomic alterations, involving the generation of new peptide sequences and taking the shape of neoantigens (Mardis, 2019). Genome instability explains tumor heterogeneity, offering critical regulation of cancer pathways, driving phenotypic variation, and impacting epigenetics modification (Burrell et al., 2013). Hence, we look forward to selecting genome instability-related biomarkers and treatment targets to shed light on LUAD immunotherapy.

lncRNA has been reported to play a direct role in regulating genome instability. *NORAD* was the first reported lncRNA maintaining genomic integrity via sequestering PUMILIO proteins (Lee et al., 2016). Munschauer et al. (2018) proved that *NORAD* controls RNA-binding motif protein X-linked (RBMX) to assemble a ribonucleoprotein complex (NORAD-activated ribonucleoprotein complex 1), mainly including suppressors of genome instability topoisomerase 1, necessary for the assembly of topoisomerase complex NARC1 (Munschauer et al., 2018; Elguindy et al., 2019). Hu et al. found that a p53-responsive lncRNA *GUARDIN* could maintain genomic integrity via the ceRNA mechanism. Therefore, we suppose a ceRNA network regulating genome instability in LUAD and GlncRs are promising immunotherapy biomarkers and treatment targets. We extracted hub-lncRNAs through WGCNA and differential expression analysis. Among the four lncRNAs (*LINC01224*, *LINC00346*, *TRPM2-AS*, and *CASC9*), *LINC01224* is primarily located in the cytoplasm, and *LINC00346* and *TRPM2-AS* are primarily located in the nucleus (the localization of CASC9 in a lung cancer cell line is unknown). lncRNA in the cytoplasm functions as ceRNA sponging miRNA and upregulates the target mRNA (Tay et al., 2014). The target mRNAs of *LINC01224* were *CCNF* and *PKMYT1*. *CCNF* belongs to the F-box protein family, which participates in the Skp1-Cul1-F-box protein (SCF) ubiquitin ligase complexes, serving as substrate recognition subunits. D’Angiolella et al. reported that *CCNF*-mediated degradation of ribonucleotide reductase family member 2 (RRM2) plays a crucial role in maintaining the balance of dNTP (which is essential for DNA synthesis and repair) levels. The alteration of dNTP levels leads to genome instability and a hypermutator phenotype (D’Angiolella et al., 2013). Besides, *CCNF* regulates the CP110 level, a centrosomal protein promoting centrosome duplication localized in the cytoplasm. Another target gene of *LINC01224*, *PKMYT1*, a “forgotten” member of the WEE kinase family, has similar functions to *WEE1*, regulating the G2–M checkpoint via cyclin-dependent kinase 1 (CDK1) phosphorylation. Notably, unlike *WEE1*, *PKMYT1* is predominantly localized in the cytoplasm and associates with the Golgi apparatus and endoplasmic reticulum through a membrane tether, where *PKMYT1* modulates *CDK1* by sequestering it in the cytoplasm (Asquith et al., 2020). Therefore, it is possible to hypothesize that *LINC01224* regulates genome instability through the *CCNF*/*RRM2* axis and *PKMYT1*/*CDK1* axis via the ceRNA mechanism. Further studies are needed to prove this point. In the nucleus, lncRNA participates in the constituents of complexes and has to do with epigenetic modulation. Although *LINC00346* and *TRPM2-AS* are predominantly localized in the nucleus, there is still a part of them that is localized in the cytoplasm, modulating genome instability via the ceRNA mechanism. In this study, we did not explore how lncRNA directly affects genome instability. In summary, we constructed a ceRNA regulatory network for genome instability in LUAD and proposed four hub-lncRNAs and seven target genes (*CCNF*, *PKMYT1*, *GCH1*, *TK1*, *PSAT1*, *ADAM33*, and *DDX11*) as potential immunotherapy biomarkers and neoadjuvant therapy treatment targets, among which *LINC01224-CCNF* and *LINC01224-PKMYT1* were the most promising axes.

We proved that the hub-lncRNAs and target mRNAs were involved in TME modulation. Immune subtypes C1–C6 mark diverse immune functions: wound healing (C1), IFN-γ dominant (C2), inflammatory (C3), lymphocyte depleted (C4), immunologically quiet (C5), and TGF-β dominant (C6) (Venteicher et al., 2017; Thorsson et al., 2018). *CCNF* had the highest expression in C4 within all six subtypes, suggesting that it was sensitive to a temporary shutdown of the naïve lymphocyte recirculation process. *CCNF* remains for further research to indicate the increase of lymphocyte numbers in responding to lymphoid organ locally or immunosuppression due to the depletion of recirculation lymphocytes systemically (Shiow et al., 2006). *PKMYT1*, *GCH1*, and *TK1* presented the highest expression in C2—IFN-γ dominant. They were biomarkers measuring inflammation. Notably, *GCH1* and *TK1* had relatively high immunophenotype expressions among all the hub-lncRNAs and target mRNAs, although they seem less valuable in other analyses. The results suggested that *GCH1* and *TK1* expressions can be used as specific indicators of impaired immune functions. Furthermore, we explored LUAD TME infiltration. Although most genes were negatively associated with immune cell infiltration, some critical immune cells that affect immunotherapy, such as CD8+ T cells and active NK cells, were positively related to these genes’ expression. Besides, most genes (6/11, 54.5%) were positively associated with *PD-L1*. These findings indicated that the 11 genes we proposed contribute to an antitumor TME infiltration, leading to tumor immune escape. To conclude, these 11 genes could be novel immunotherapy biomarkers on the aspects of LUAD genome instability and immune features.

Genome instability is associated with DNA replication, the most vulnerable cellular process, and is often accompanied by increased tumor heterogeneity. Severe DNA damage leads to replication stress, which is a feature of pre-cancer and cancer cells and provides a source of genome instability. Tumors are a patchwork of cells with diverse capacities of self-renewal, tumorigenicity, and differentiation potential with hierarchical organization, and intratumor heterogeneity offers the fuel. Cancer stem cell (CSC) plays a driver role in intratumor heterogeneity (Kreso and Dick, 2014). High cancer stemness is usually associated with an increased mutation load and provides a strong hint of a “cold” immune microenvironment, resulting in immune suppression (Miranda et al., 2019). The clinical value of CSC is intriguing, while the sensitivity of current biomarkers used for monitoring CSCs, including *CD133*, *CD44*, and aldehyde dehydrogenase (*ALDH*), is not guaranteed in LUAD (Skvortsov et al., 2018). In this study, we found that most genes were positively related to cancer stemness (10/11, 90.9%), among which the correlation between *CCNF*, *PKMYT1*, *TK1*, and RNAss reached 0.5 (*P* < 0.001), which is consistent with previous literature, and these genes could be cancer stemness and tumor heterogeneity biomarkers. Besides, only *ADAM33* was negatively related to cancer stemness, still needing further research. Tumor heterogeneity is a significant cause of drug resistance, caused by the expansion of specific drug-tolerant subclonal populations or the evolution of novel drug-tolerant cells under selective therapeutic pressure (Dagogo-Jack and Shaw, 2018). Our drug sensitivity analysis is consistent with this hypothesis. We found that some protein kinase-targeted drugs, such as dasatinib and cobimetinib, and genome instability-related genes were negatively related to sensitivity, which the high tumor heterogeneity could explain. In contrast, the sensitivity of drugs that affect DNA replication and synthesis, such as 5-fluorodeoxyuridine 10mer, pyrazoloacridine, and palbociclib, was positively related to these 11 genes. These findings provide a new perspective for genomic instability and emphasize the clinical significance of genomic instability in tumor treatment.

There are still some limitations in this study. First, we did not explore how lncRNA directly affects genome instability. Second, the potential mechanisms underlying our findings still need biological validation through *in vitro* and *in vivo* experiments. Third, due to the diversity of lncRNA sequencing techniques, we did not find an appropriate testing cohort independent of the TCGA cohort to validate our clustering and signature further. Nevertheless, we tried our best to minimize the selection bias, including random division and the chi-square test.

To conclude, we performed an integrative multi-omics analysis to explore the mechanisms and clinical value of genome instability-related lncRNA in LUAD. We discovered that sponging miRNA, genome instability-related lncRNA functions as ceRNA, modulating genomic integrity. This research provides clinical references for LUAD immunotherapy and prognosis and interprets a potential genome instability-related ceRNA regulatory network in which *LINC01224*-*miR-485-5p*/*miR-29c-3p*-*CCNF*-*RRM2* and *LINC01224*-*miR485-5p*-*PKMYT1*-*CDK1* axes were the most promising pathways.

## Data Availability Statement

The datasets presented in this study can be found in online repositories. The names of the repository/repositories and accession number(s) can be found in the article/Supplementary Material.

## Author Contributions

ZW and ZR performed the data analysis and wrote the first draft. RL, JG, and YX revised the manuscript and carried out data collection. JG and GZ revised the manuscript and drafted the tables. YQ designed and supervised the study. All authors contributed to the article and approved the submitted version.

## Conflict of Interest

The authors declare that the research was conducted in the absence of any commercial or financial relationships that could be construed as a potential conflict of interest.

## Footnotes

1 https://www.ncbi.nlm.nih.gov/assembly/GCF_000001405.39/

2 http://lncatlas.crg.eu/

3 https://xenabrowser.net/datapages/

4 http://gepia.cancer-pku.cn/about.html

5 https://discover.nci.nih.gov/cellminer/

6 http://bioinformatics.mdanderson.org/estimate/

## References

[B1] Asquith C. R. M. Laitinen T. East M. P. (2020). PKMYT1: a forgotten member of the WEE1 family. *Nat. Rev. Drug Discov.* 19:157. 10.1038/d41573-019-00202-9 32127662

[B2] Burrell R. A. McGranahan N. Bartek J. Swanton C. (2013). The causes and consequences of genetic heterogeneity in cancer evolution. *Nature* 501 338–345. 10.1038/nature12625 24048066

[B3] Carbone D. P. Reck M. Paz-Ares L. Creelan B. Horn L. Steins M. (2017). First-line nivolumab in stage IV or recurrent non-small-cell lung cancer. *N. Engl. J. Med.* 376 2415–2426. 10.1056/NEJMoa1613493 28636851 PMC6487310

[B4] Cerami E. Gao J. Dogrusoz U. Gross B. E. Sumer S. O. Aksoy B. A. (2012). The cBio cancer genomics portal: an open platform for exploring multidimensional cancer genomics data. *Cancer Discov.* 2 401–404. 10.1158/2159-8290.Cd-12-0095 22588877 PMC3956037

[B5] Chen L.-L. (2016). Linking long noncoding RNA localization and function. *Trends Biochem. Sci.* 41 761–772. 10.1016/j.tibs.2016.07.003 27499234

[B6] D’Angiolella V. Esencay M. Pagano M. (2013). A cyclin without cyclin-dependent kinases: cyclin F controls genome stability through ubiquitin-mediated proteolysis. *Trends Cell Biol.* 23 135–140. 10.1016/j.tcb.2012.10.011 23182110 PMC3597434

[B7] Dagogo-Jack I. Shaw A. T. (2018). Tumour heterogeneity and resistance to cancer therapies. *Nat. Rev. Clin. Oncol.* 15 81–94. 10.1038/nrclinonc.2017.166 29115304

[B8] Elguindy M. M. Kopp F. Goodarzi M. Rehfeld F. Thomas A. Chang T. C. (2019). PUMILIO, but not RBMX, binding is required for regulation of genomic stability by noncoding RNA NORAD. *Elife* 8:e48625. 10.7554/eLife.48625 31343408 PMC6677556

[B9] Frost N. Kollmeier J. Misch D. Vollbrecht C. Grah C. Matthes B. (2021). Pembrolizumab as first-line palliative therapy in PD-L1 overexpressing (= 50%) NSCLC: real-world results with special focus on PS = 2, brain metastases, and steroids. *Clin. Lung Cancer.* 10.1016/j.cllc.2021.02.001 [Epub ahead of print]. 33648877

[B10] Hu W. L. Jin L. Xu A. Wang Y. F. Thorne R. F. Zhang X. D. (2018). GUARDIN is a p53-responsive long non-coding RNA that is essential for genomic stability. *Nat. Cell Biol.* 20 492–502. 10.1038/s41556-018-0066-7 29593331

[B11] Kreso A. Dick J. E. (2014). Evolution of the cancer stem cell model. *Cell Stem Cell* 14 275–291. 10.1016/j.stem.2014.02.006 24607403

[B12] Lee J. J. Park S. Park H. Kim S. Lee J. Lee J. (2019). Tracing oncogene rearrangements in the mutational history of lung adenocarcinoma. *Cell* 177 1842–1857.e21. 10.1016/j.cell.2019.05.013 31155235

[B13] Lee S. Kopp F. Chang T. C. Sataluri A. Chen B. Sivakumar S. (2016). Noncoding RNA NORAD regulates genomic stability by sequestering PUMILIO proteins. *Cell* 164 69–80. 10.1016/j.cell.2015.12.017 26724866 PMC4715682

[B14] Li F. Huang Q. Luster T. A. Hu H. Zhang H. Ng W. L. (2020). In vivo epigenetic CRISPR screen identifies Asf1a as an immunotherapeutic target in Kras-mutant lung adenocarcinoma. *Cancer Discov.* 10 270–287. 10.1158/2159-8290.Cd-19-0780 31744829 PMC7007372

[B15] Li J. Ge J. Yang Y. Liu B. Zheng M. Shi R. (2020). Long noncoding RNA ZFPM2-AS1 is involved in lung adenocarcinoma via miR-511-3p/AFF4 pathway. *J. Cell. Biochem.* 121 2534–2542. 10.1002/jcb.29476 31692047

[B16] Li J.-H. Liu S. Zhou H. Qu L.-H. Yang J.-H. (2013). starBase v2.0: decoding miRNA-ceRNA, miRNA-ncRNA and protein–RNA interaction networks from large-scale CLIP-Seq data. *Nucleic Acids Res.* 42 D92–D97. 10.1093/nar/gkt1248 24297251 PMC3964941

[B17] Liu L. Bai X. Wang J. Tang X. R. Wu D. H. Du S. S. (2019). Combination of TMB and CNA stratifies prognostic and predictive responses to immunotherapy across metastatic cancer. *Clin. Cancer Res.* 25 7413–7423. 10.1158/1078-0432.Ccr-19-0558 31515453

[B18] Malta T. M. Sokolov A. Gentles A. J. Burzykowski T. Poisson L. Weinstein J. N. (2018). Machine learning identifies stemness features associated with oncogenic dedifferentiation. *Cell* 173 338–354.e15. 29625051 10.1016/j.cell.2018.03.034 PMC5902191

[B19] Mardis E. R. (2019). Neoantigens and genome instability: impact on immunogenomic phenotypes and immunotherapy response. *Genome Med.* 11:71. 10.1186/s13073-019-0684-0 31747945 PMC6865009

[B20] Miranda A. Hamilton P. T. Zhang A. W. Pattnaik S. Becht E. Mezheyeuski A. (2019). Cancer stemness, intratumoral heterogeneity, and immune response across cancers. *Proc. Natl. Acad. Sci. U.S.A.* 116 9020–9029. 10.1073/pnas.1818210116 30996127 PMC6500180

[B21] Mok T. S. K. Wu Y. L. Kudaba I. Kowalski D. M. Cho B. C. Turna H. Z. (2019). Pembrolizumab versus chemotherapy for previously untreated, PD-L1-expressing, locally advanced or metastatic non-small-cell lung cancer (KEYNOTE-042): a randomised, open-label, controlled, phase 3 trial. *Lancet* 393 1819–1830. 10.1016/s0140-6736(18)32409-7 30955977

[B22] Morel A. P. Ginestier C. Pommier R. M. Cabaud O. Ruiz E. Wicinski J. (2017). A stemness-related ZEB1-MSRB3 axis governs cellular pliancy and breast cancer genome stability. *Nat. Med.* 23 568–578. 10.1038/nm.4323 28394329

[B23] Munschauer M. Nguyen C. T. Sirokman K. Hartigan C. R. Hogstrom L. Engreitz J. M. (2018). The NORAD lncRNA assembles a topoisomerase complex critical for genome stability. *Nature* 561 132–136. 10.1038/s41586-018-0453-z 30150775

[B24] Niu Y. Lin A. Luo P. Zhu W. Wei T. Tang R. (2020). Prognosis of lung adenocarcinoma patients with NTRK3 mutations to immune checkpoint inhibitors. *Front. Pharmacol.* 11:1213. 10.3389/fphar.2020.01213 32903385 PMC7434857

[B25] Overman M. J. McDermott R. Leach J. L. Lonardi S. Lenz H. J. Morse M. A. (2017). Nivolumab in patients with metastatic DNA mismatch repair-deficient or microsatellite instability-high colorectal cancer (CheckMate 142): an open-label, multicentre, phase 2 study. *Lancet Oncol.* 18 1182–1191. 10.1016/s1470-2045(17)30422-9 28734759 PMC6207072

[B26] Peyraud F. Italiano A. (2020). Combined PARP inhibition and immune checkpoint therapy in solid tumors. *Cancers* 12:1502. 10.3390/cancers12061502 32526888 PMC7352466

[B27] Pros E. Saigi M. Alameda D. Gomez-Mariano G. Martinez-Delgado B. Alburquerque-Bejar J. J. (2020). Genome-wide profiling of non-smoking-related lung cancer cells reveals common RB1 rearrangements associated with histopathologic transformation in EGFR-mutant tumors. *Ann. Oncol.* 31 274–282. 10.1016/j.annonc.2019.09.001 31959344

[B28] R Core Team (2013). *R: A Language and Environment for Statistical Computing.* Vienna: R Foundation for Statistical Computing.

[B29] Rittmeyer A. Barlesi F. Waterkamp D. Park K. Ciardiello F. von Pawel J. (2017). Atezolizumab versus docetaxel in patients with previously treated non-small-cell lung cancer (OAK): a phase 3, open-label, multicentre randomised controlled trial. *Lancet* 389 255–265. 10.1016/s0140-6736(16)32517-x 27979383 PMC6886121

[B30] Rizvi H. Sanchez-Vega F. La K. Chatila W. Jonsson P. Halpenny D. (2018). Molecular determinants of response to anti-programmed cell death (PD)-1 and anti-programmed death-ligand 1 (PD-L1) blockade in patients with non-small-cell lung cancer profiled with targeted next-generation sequencing. *J. Clin. Oncol.* 36 633–641. 10.1200/jco.2017.75.3384 29337640 PMC6075848

[B31] Salmena L. Poliseno L. Tay Y. Kats L. Pandolfi P. P. (2011). A ceRNA hypothesis: the Rosetta stone of a hidden RNA language? *Cell* 146 353–358. 10.1016/j.cell.2011.07.014 21802130 PMC3235919

[B32] Sansregret L. Vanhaesebroeck B. Swanton C. (2018). Determinants and clinical implications of chromosomal instability in cancer. *Nat. Rev. Clin. Oncol.* 15 139–150. 10.1038/nrclinonc.2017.198 29297505

[B33] Shannon P. Markiel A. Ozier O. Baliga N. S. Wang J. T. Ramage D. (2003). Cytoscape: a software environment for integrated models of biomolecular interaction networks. *Genome Res.* 13 2498–2504. 10.1101/gr.1239303 14597658 PMC403769

[B34] Shiow L. R. Rosen D. B. Brdicková N. Xu Y. An J. Lanier L. L. (2006). CD69 acts downstream of interferon-alpha/beta to inhibit S1P1 and lymphocyte egress from lymphoid organs. *Nature* 440 540–544. 10.1038/nature04606 16525420

[B35] Skvortsov S. Skvortsova I. I. Tang D. G. Dubrovska A. (2018). Concise review: prostate cancer stem cells: current understanding. *Stem Cells* 36 1457–1474. 10.1002/stem.2859 29845679 PMC7903656

[B36] Sun J. Zhang Z. Bao S. Yan C. Hou P. Wu N. (2020). Identification of tumor immune infiltration-associated lncRNAs for improving prognosis and immunotherapy response of patients with non-small cell lung cancer. *J. Immunother. Cancer* 8:e000110. 10.1136/jitc-2019-000110 32041817 PMC7057423

[B37] Takamochi K. Takahashi F. Suehara Y. Sato E. Kohsaka S. Hayashi T. (2017). DNA mismatch repair deficiency in surgically resected lung adenocarcinoma: microsatellite instability analysis using the Promega panel. *Lung Cancer* 110 26–31. 10.1016/j.lungcan.2017.05.016 28676214

[B38] Tanvetyanon T. Creelan B. C. Antonia S. J. (2016). The safety and efficacy of nivolumab in advanced (metastatic) non-small cell lung cancer. *Expert Rev. Anticancer Ther.* 16 903–910. 10.1080/14737140.2016.1220836 27488231

[B39] Tay Y. Rinn J. Pandolfi P. P. (2014). The multilayered complexity of ceRNA crosstalk and competition. *Nature* 505 344–352. 10.1038/nature12986 24429633 PMC4113481

[B40] Thorsson V. Gibbs D. L. Brown S. D. Wolf D. Bortone D. S. Ou Yang T. H. (2018). The immune landscape of cancer. *Immunity* 48 812–830.e14. 10.1016/j.immuni.2018.03.023 29628290 PMC5982584

[B41] Tubbs A. Nussenzweig A. (2017). Endogenous DNA damage as a source of genomic instability in cancer. *Cell* 168 644–656. 10.1016/j.cell.2017.01.002 28187286 PMC6591730

[B42] Venteicher A. S. Tirosh I. Hebert C. Yizhak K. Neftel C. Filbin M. G. (2017). Decoupling genetics, lineages, and microenvironment in IDH-mutant gliomas by single-cell RNA-seq. *Science* 355:eaai8478. 10.1126/science.aai8478 28360267 PMC5519096

[B43] Ventura A. (2016). NORAD: defender of the genome. *Trends Genet.* 32 390–392. 10.1016/j.tig.2016.04.002 27157388

[B44] Wang F. Zhao Q. Wang Y. N. Jin Y. He M. M. Liu Z. X. (2019). Evaluation of POLE and POLD1 mutations as biomarkers for immunotherapy outcomes across multiple cancer types. *JAMA Oncol.* 5 1504–1506. 10.1001/jamaoncol.2019.2963 31415061 PMC6696731

[B45] Wang L. Liang Y. Mao Q. Xia W. Chen B. Shen H. (2019). Circular RNA circCRIM1 inhibits invasion and metastasis in lung adenocarcinoma through the microRNA (miR)-182/miR-93-leukemia inhibitory factor receptor pathway. *Cancer Sci.* 110 2960–2972. 10.1111/cas.14131 31301086 PMC6726696

[B46] Yoshihara K. Shahmoradgoli M. Martínez E. Vegesna R. Kim H. Torres-Garcia W. (2013). Inferring tumour purity and stromal and immune cell admixture from expression data. *Nat. Commun.* 4:2612. 10.1038/ncomms3612 24113773 PMC3826632

[B47] Yu Y. Zeng D. Ou Q. Liu S. Li A. Chen Y. (2019). Association of survival and immune-related biomarkers with immunotherapy in patients with non-small cell lung cancer: a meta-analysis and individual patient-level analysis. *JAMA Netw. Open* 2:e196879. 10.1001/jamanetworkopen.2019.6879 31290993 PMC6625073

[B48] Zhang X. Klamer B. Li J. Fernandez S. Li L. (2020). A pan-cancer study of class-3 semaphorins as therapeutic targets in cancer. *BMC Med. Genomics* 13(Suppl. 5):45. 10.1186/s12920-020-0682-5 32241267 PMC7118829

[B49] Zhong Y. Wang J. Lv W. Xu J. Mei S. Shan A. (2019). LncRNA TTN-AS1 drives invasion and migration of lung adenocarcinoma cells via modulation of miR-4677-3p/ZEB1 axis. *J. Cell. Biochem.* 120 17131–17141. 10.1002/jcb.28973 31173403

[B50] Zhou G. W. Xiong Y. Chen S. Xia F. Li Q. Hu J. (2016). Anti-PD-1/PD-L1 antibody therapy for pretreated advanced nonsmall-cell lung cancer: a meta-analysis of randomized clinical trials. *Medicine* 95:e4611. 10.1097/md.0000000000004611 27583876 PMC5008560

