# Supplementary figures and images for: Multi-Omics Integrative Bioinformatics Analyses Reveal Long Non-coding RNA Modulates Genomic Integrity via Competing Endogenous RNA Mechanism and Serves as Novel Biomarkers for Overall Survival in Lung Adenocarcinoma
Source: Front Cell Dev Biol. 2021 Jul 22;9:691540. doi: 10.3389/fcell.2021.691540 (PMC8339593; doi:10.3389/fcell.2021.691540)

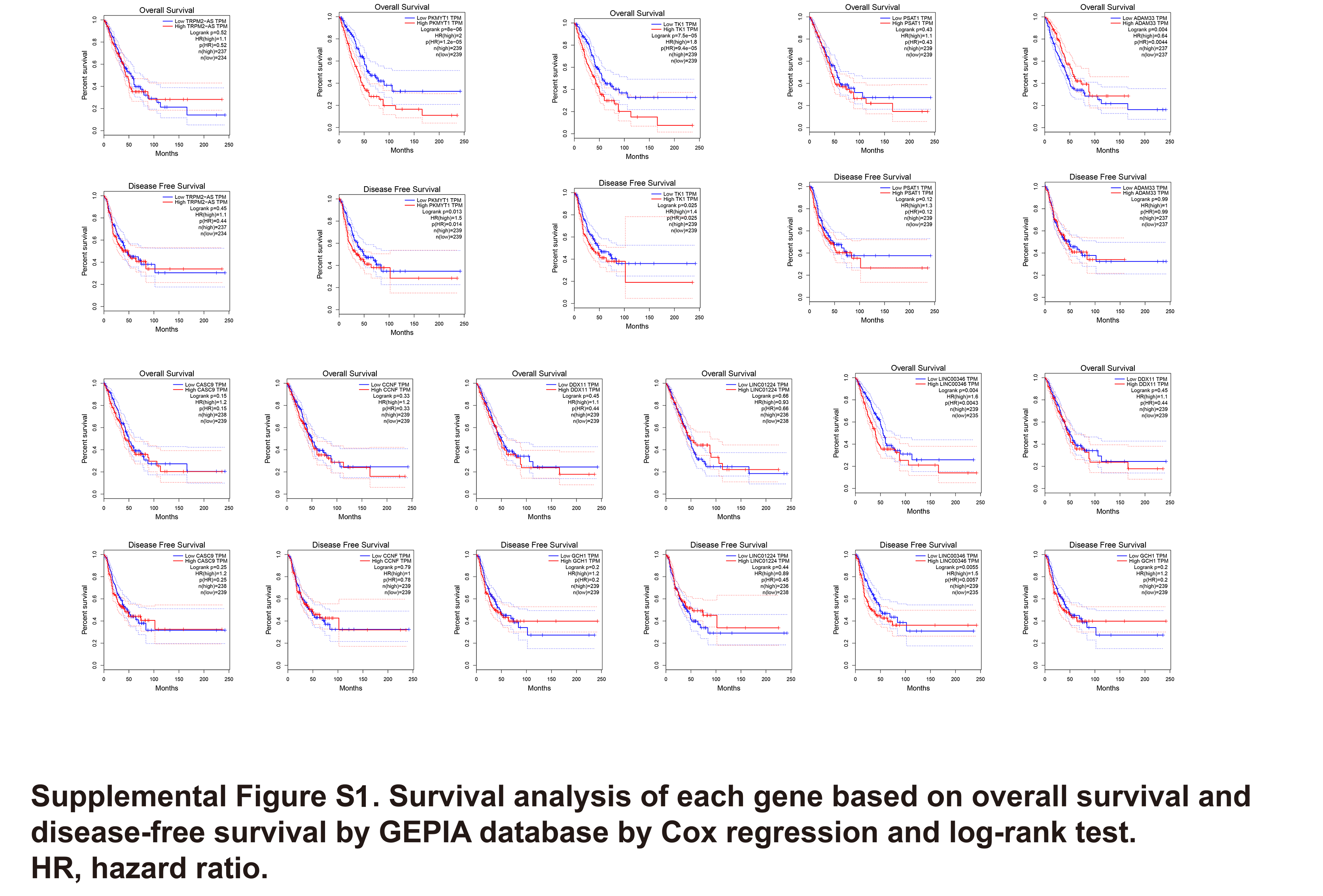

Supplement: Supplementary file 1 [file Image_1.TIF]

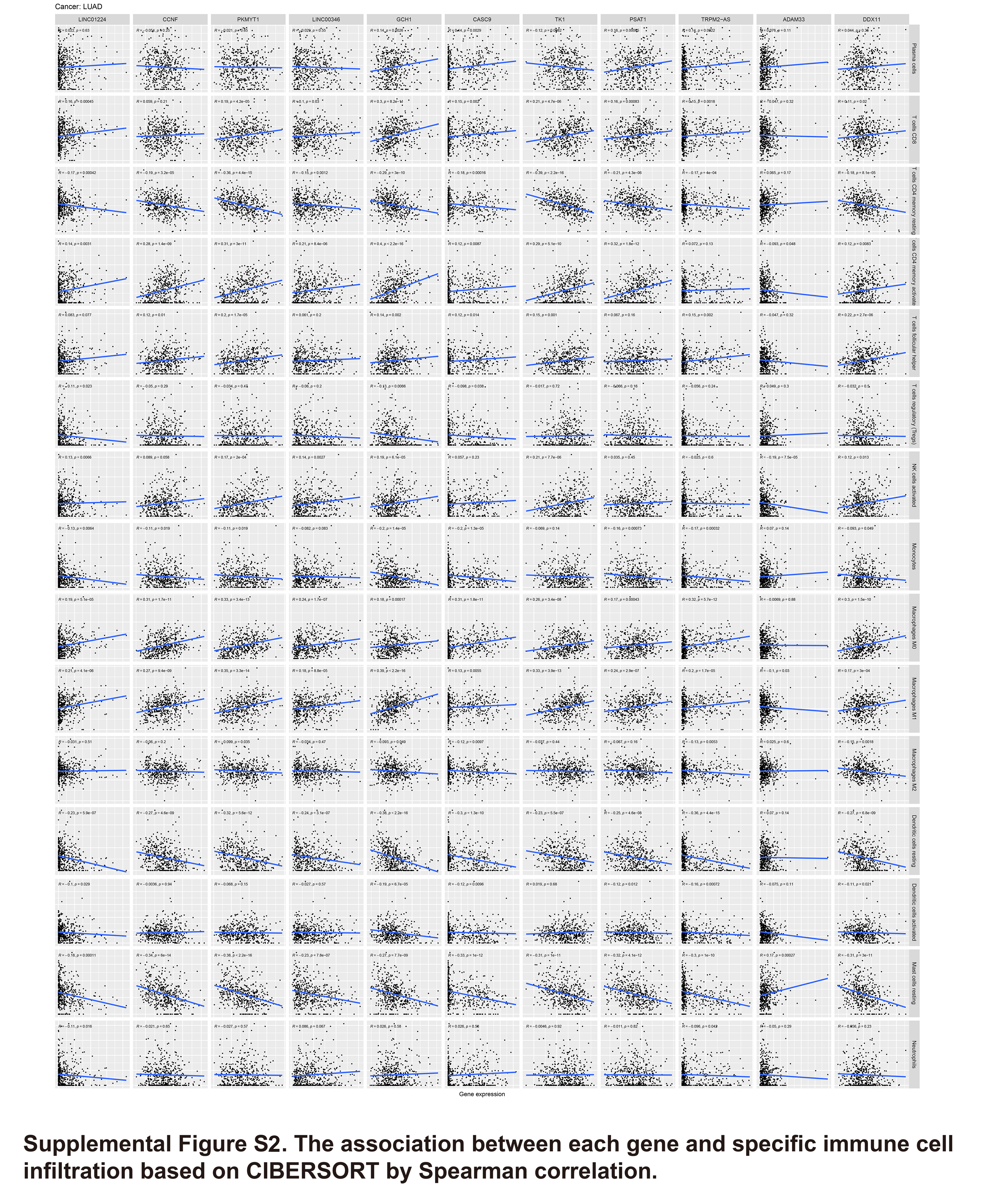

Supplement: Supplementary file 2 [file Image_2.TIF]

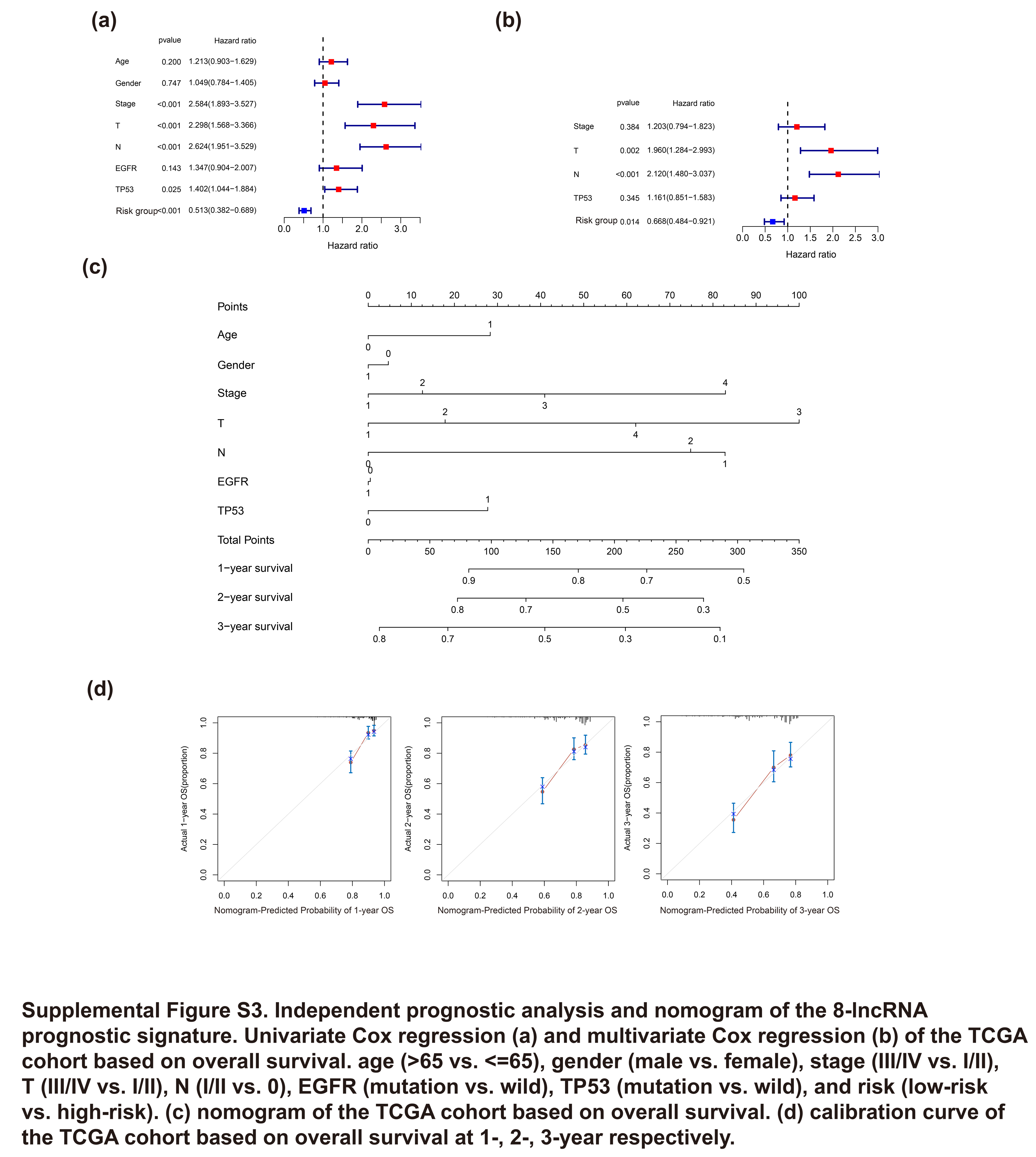

Supplement: Supplementary file 3 [file Image_3.TIF]
